# DNA barcoding reveals that the common cupped oyster in Taiwan is the Portuguese oyster *Crassostrea angulata* (Ostreoida; Ostreidae), not *C. gigas*

**DOI:** 10.1038/srep34057

**Published:** 2016-09-26

**Authors:** Sheng-Tai Hsiao, Shin-Chang Chuang, Kao-Sung Chen, Ping-Ho Ho, Chi-Lun Wu, Chaolun Allen Chen

**Affiliations:** 1 Fisheries Research Institute, Council of Agriculture, Keelung, 20246, Taiwan; 2 Department of Environment Biology and Fisheries Science, National Taiwan Ocean University, Keelung, 20224, Taiwan; 3 Biodiversity Research Center, Academia Sinica, Nangang, Taipei, 11574, Taiwan; 4 Taiwan International Graduate Program-Biodiversity, Academia Sinica, Nangang, Taipei, 11574, Taiwan; 5 National Taiwan University, Institute of Oceanography, Taipei 10617, Taiwan

## Abstract

The Pacific cupped oyster, *Crassostrea gigas,* is one of the major aquacultural shellfish species that has been introduced to Europe and America from its native source in the West Pacific. In Taiwan, the cultivated cupped oysters along the west coast have been identified as *C. gigas* for over centuries; however, several molecular phylogenetic studies have cast doubt upon the existence of this species in Taiwan and adjacent waters. Indeed, our analyses of mitochondrial cytochrome oxidase I (COI) sequences from 313 *Crassostrea* collected from 12 locations along Taiwanese and southern Chinese coastlines confirm that all samples were the Portuguese oyster, *C. angulata*, rather than *C. gigas.* Multiple lines of evidence, including haplotypic and nucleotide diversity of the COI gene, demographic history, and population genetics, suggest that Taiwanese *C. angulata* is unique, probably experienced a sudden population expansion after the Last Glacial Maxima around 20,000 years ago, and has a significantly limited genetic connectivity across the Taiwan Strait. Our study applies an extended sampling and DNA barcoding to confirm the absence of *C. gigas* in natural and cultivated populations in Taiwan and southern China, where we only found *C. angulata*. We highlight the importance of conserving the gene pool of the *C. angulata* population in Taiwan, particularly considering the current threats by large-scale environmental disturbances such as marine pollution, habitat destruction, and climate change.

Cupped oysters belonging to the genus *Crassostrea* are among the most important cultivated shellfish species in the world^1^. Among the three species of *Crassostrea* listed by the FAO, the Pacific cupped oyster, *C. gigas*, contributed an estimated 555k tons to global aquaculture production in 2013^1^. Taiwan’s cultivated cupped oyster species has been identified as *C. gigas* for over a century and has produced 27,793 tons in 2013 having an economic value of over US$20 million^2^. However, molecular phylogenetic analyses of *Crassostrea* species have raised concern over the taxonomic status of *C. gigas* in Taiwan^3^,^4^,^5^,^6^,^7^,^8^,^9^. Molecular phylogenetic studies on the origin of the Portuguese oyster, *C. angulata,* using small numbers of Taiwanese cupped oysters as the Asian reference, unexpectedly showed that those from Taiwan were grouped with *C. angulata* from the Atlantic^3^,^4^ instead of being clustered with *C. gigas* from Japan. *C. angulata* is a species assumed to be native to the northeastern Atlantic but is morphologically and enzymatically indistinguishable from *C. gigas*^5^,^6^,^10^. In contrast, karyotype analysis highlighted the close genetic similarity of these two taxa in comparison with other cupped oyster species^11^, but distinct differences between their respective karyotypes were also observed on certain chromosome pair and restriction enzyme ideograms^12^,^13^,^14^. Divergence time estimation based on the completed mitochondrial genomes of *Crassostrea* oysters confirmed that *C. gigas* and *C. angulata* are closely-related and probably diverged about 2.7 million years ago (mya)^15^,^16^. Subsequent sampling along the Chinese coast demonstrates that *C. angulata* has a wide distribution in southern China, with the Yangtze River separating it from *C. gigas* in northern China^8^,^9^. Taiwan, the largest continental island situated at the border between the northern and southern Chinese coasts, possesses climates ranging from tropical to subtropical/temperate. The Taiwanese coast provides a variety of marine environments that host a relatively highly diverse fauna^17^,^18^, thus *C. angulata* and *C. gigas* might prove to coexist along the Taiwan coast if intensively sampled. In addition, geological events such as the last glacial maximum (LGM~20 ka BP) are speculated to have affected the phylogeography of marine biota in the western Pacific^19^,^20^,^21^ and might play an important role in shaping the demographic history of *Crassostrea* species in Taiwan.

DNA barcoding based on the mitochondrial cytochrome oxidase I (COI) fragment has been a standard way to discriminate between closely related species, identify new, cryptic, or invasive species, and assess species assemblages of communities across many animal phyla. The general rule in DNA barcoding is that intraspecific COI variation is <1%, whereas interspecific divergence is normally >2%^22^. Here, we applied DNA barcoding of a COI fragment to examine the status of cultivated *Crassostrea* species from eight populations in Taiwan and four along the southern China coast (Supplementary Fig. 1S). In addition, the published COI DNA sequences of *C. gigas* from the northwestern Pacific (China, Japan, and Korea) were retrieved from GenBank^20^ for phylogenetic and phylogeographic comparisons.

## Results

**Figure 1:**
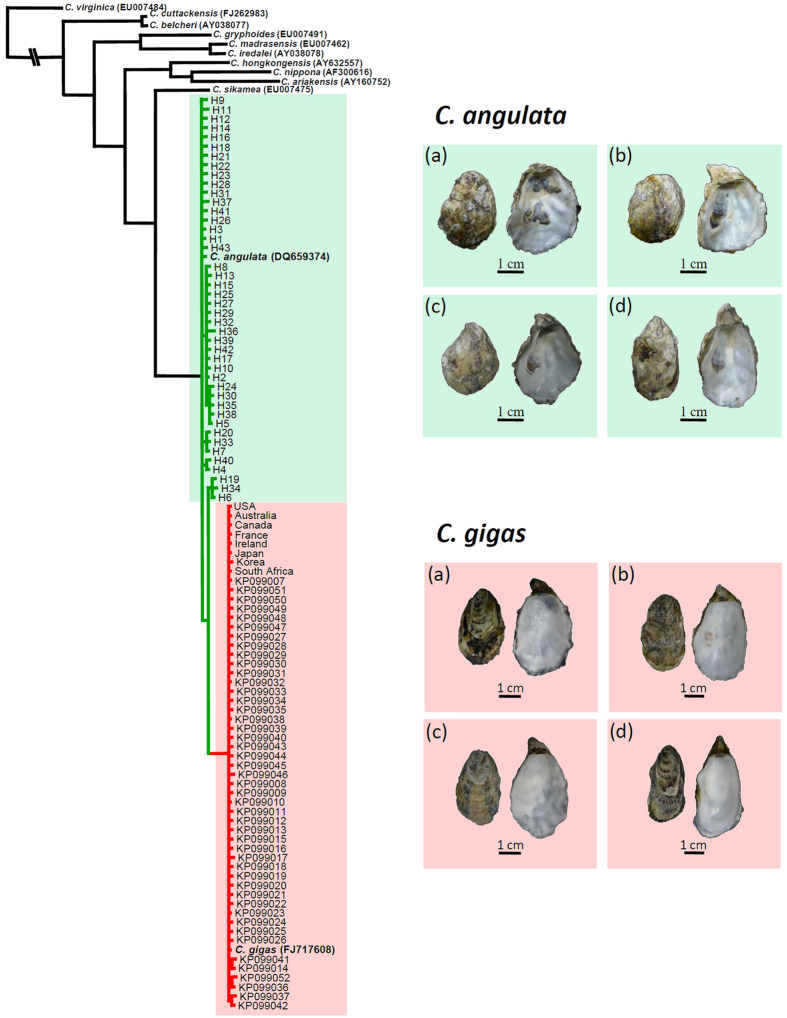
Phylogenetic tree constructed for Taiwan *Crassostrea* oysters and reference sequences based on the COI gene. Branch support values estimated by bootstrap pseudo-replicates in maximum-likelihood and Bayesian inference, respectively, are shown above each branch. A minus sign (−) indicates that bootstrap values and Bayesian posterior probabilities were <75%. 46 sequences of *C. gigas* from the northwestern Pacific were retrieved from GenBank^20^ (KP099007- KP099052). The accompanied photos showed the shell morphology of these two species used in this study. *C. angulata* were collected from (**a**) Hsinchu, (**b**) Changhua, (C) Tainan, and (**d**) Pingtung in Taiwan. *C. gigas* purchased from market were imported from (**a**) Australia, (**b**) France, (**c**) South Africa, (**d**) and Ireland to Taiwan.

We obtained COI sequences from 313 *Crassostrea* samples collected from 12 locations along the Taiwanese and Chinese coasts. In a total of 43 haplotypes with 41 variable sites, 14 phylogenetically informative sites were identified for *Crassostrea*. The COI phylogenies constructed using ML and Bayesian methods produced identical topologies (Fig. 1). Instead of grouping with *C. gigas,* all 43 COI haplotypes formed a monophyletic group with the Portuguese oyster, *C. angulata* (DQ659374), with high bootstrap support (99) and Bayesian posterior probability (100). ITS-1 phylogeny showed that all oyster samples collected in this study were of *C. angulata*, not *C. gigas* (Supplementary Fig. 2S). Our results confirmed that *C. gigas* does not occur along the coasts of Taiwan and southern China, regardless of whether the oysters were from marine farms or natural populations. Therefore, *C. angulata* was used herein as the species epithet in the following analyses to infer phylogenetic, population genetic structure, and demographic history.

The nucleotide composition of the COI gene fragment in *C. angulata* was A + T-rich (A, 22.4%; T, 38%), and variations consisted predominantly of transition substitutions (Ti: Tv = 11.78). The number of haplotypes (n_*h*_) ranged from 4 in Beihai to 14 in Chiayi. The number of endemic haplotypes (n_*eh*_) was significantly higher in populations along the Taiwanese coast than from the Chinese coast (X^2^-test = 72.921, p < 0.001). Haplotypic diversity (*h*) was high in all populations, with a mean value of 0.882 ± 0.012. In contrast, nucleotide diversity (π) was low, with a mean value of 0.00396 ± 0.00017 (Table 1).

**Figure 2:**
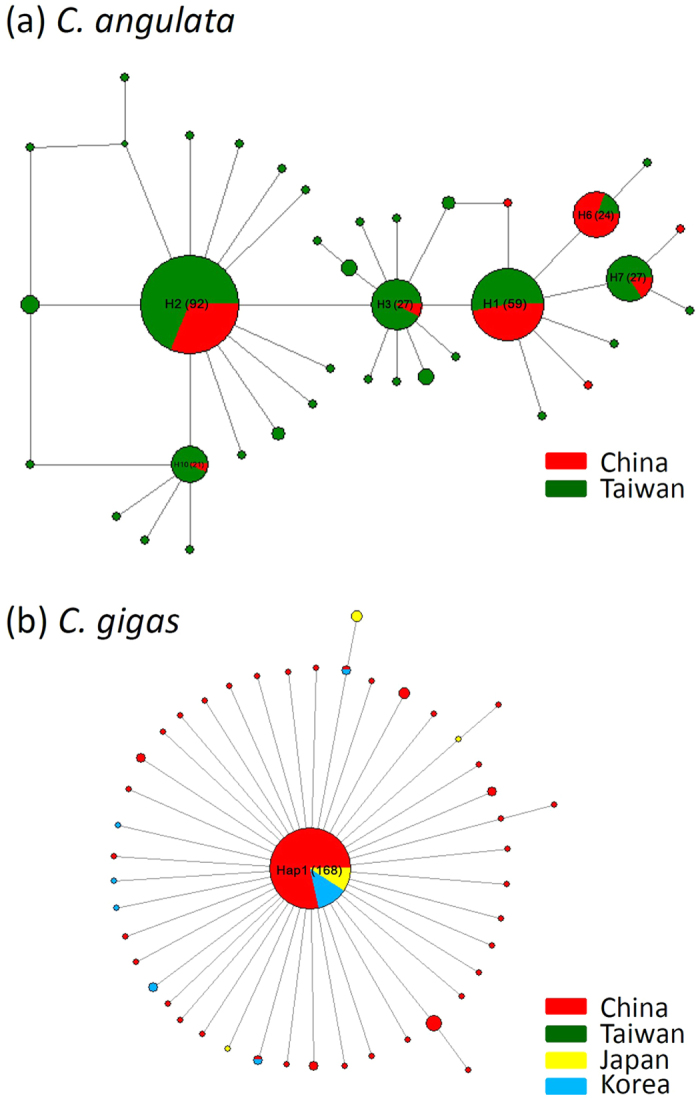
Median-joining networks and sampling sites of *Crassostrea angulata* and *Crassostrea gigas*. Circle sizes are proportional to haplotype frequencies. Different colors indicate haplotypes in corresponding regions.

**Figure 3:**
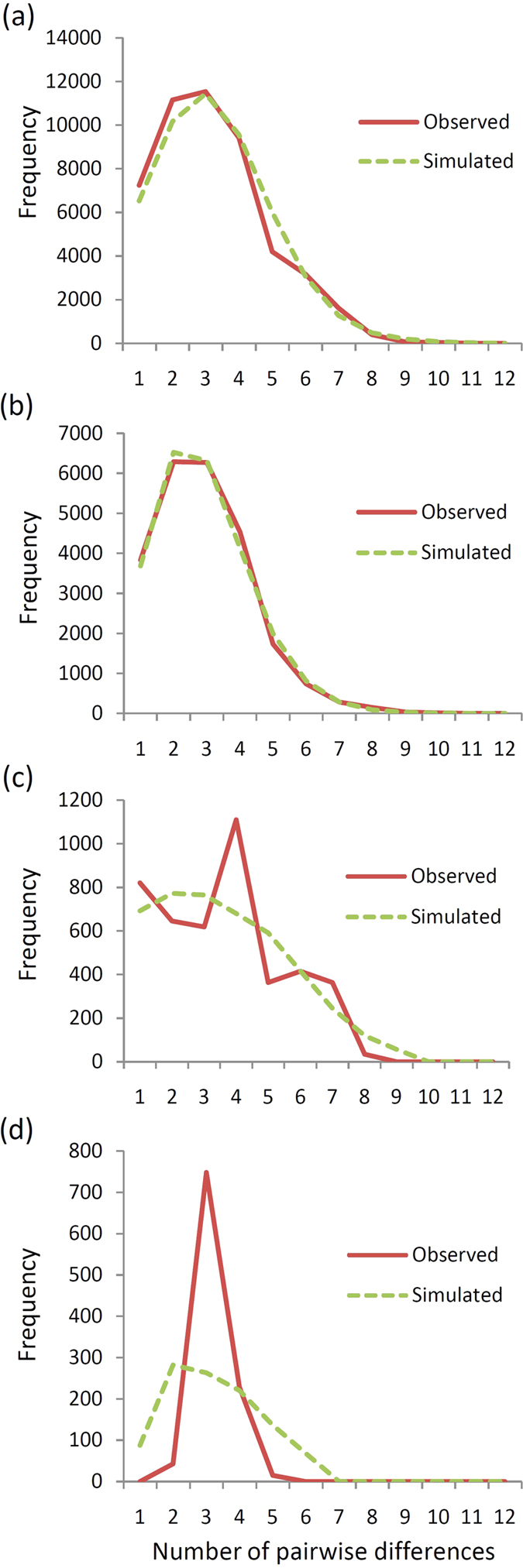
Mismatch distribution for the mtCOI of (**a**) overall *Crassostrea angulata*; (**b**) *C. angulata*, of Taiwanese coast; (**c**) *C. angulata*, of Chinese coast; (**d**) *C. gigas*^20^. The *bar* represents observed frequency and the *line* represents modeled frequency.

**Figure 4:**
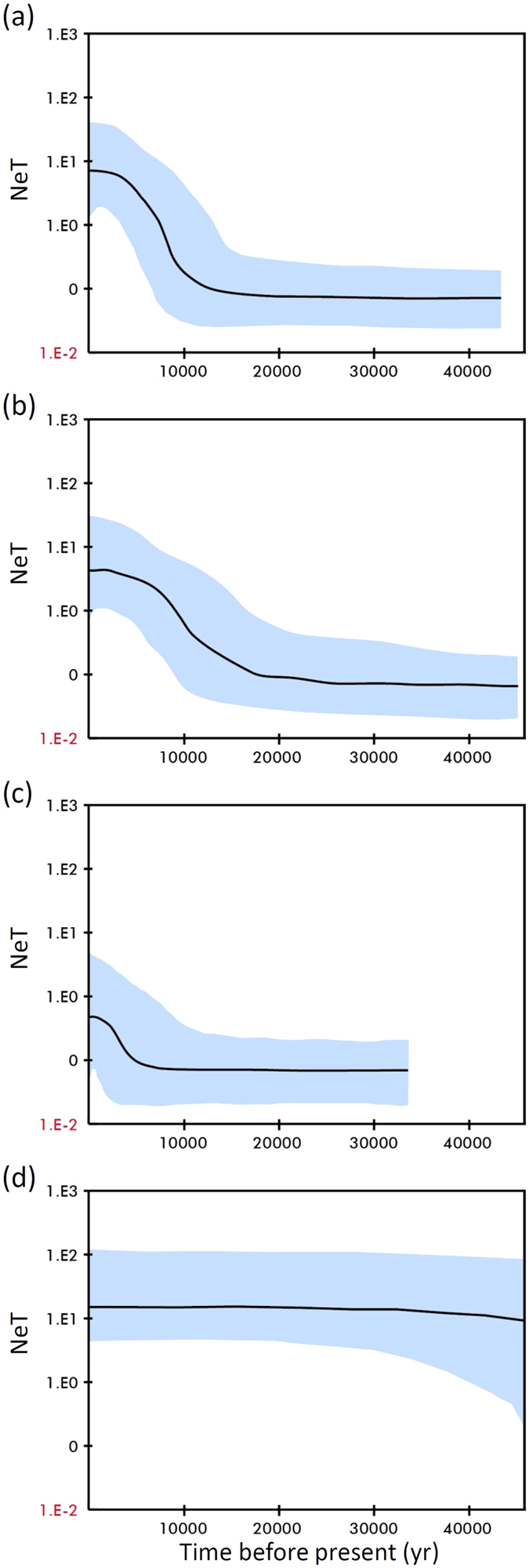
Bayesian skyline plotting of (**a**) overall *Crassostrea angulata*; (**b**) *C. angulata*, of Taiwanese coast; (**c**) *C. angulata*, of Chinese coast; (**d**) *C. gigas*^20^.The Bayesian skyline plot is derived from mtDNA COI sequences, where the x-axis is time in years and the y-axis is the product of effective population size (Ne) and generation time (T). The black line indicates the median estimate, and dashed lines indicate the 95% highest posterior density (HPD) region.

Relationships between haplotypes are represented on a median-joining network (MJN). The MJN analysis showed a complex star-like network of six haplotypes accounted for 79.87% of the COI DNA sequences collected from *C. angulata* (Fig. 2a). In contrast, the MJN of *C. gigas* was a typical star-like network dominated by one haplotype (Fig. 2b). The MJN showed no obvious haplotype clustering with respect to Taiwanese and Chinese coast populations. The high mean haplotypic diversity (0.882 ± 0.012) relative to low mean nucleotide diversity (0.00396 ± 0.00017) in *C. angulata* is indicative of a population bottleneck followed by rapid population growth and accumulation of mutations^23^,^24^. Summary statistics for testing drift-mutation equilibrium (Tajima’s D and *Fu’* Fs) showed that the two *C. angulata* populations along the Taiwanese and Chinese coasts experienced different demographic histories (Table 2). The Tajima’s D (−2.03279) and Fu’s F*s* (−27.30487) values along the Taiwanese coast were significantly negative (*p* < 0.05), indicating these populations had a sudden population expansion, although most individuals were not statically significant in these two parameters. In contrast, Tajima’s D (0.01183) was positive and Fu’s F_s_ (−1.11287) along the Chinese coast was not significantly negative, suggesting that these populations have suffered a recent bottleneck or expansion. The different demographic history across the two sides of the Taiwan Strait is also illustrated by mismatched distributions (Fig. 3). The combined data from Chinese and Taiwanese populations showed a unimodal frequency distribution similar to an analysis of the Taiwan-only population (Fig. 3a,b). In contrast, the Chinese population had a negative binomial frequency distribution (Fig. 3c). Tau values (τ = 2.865) can provide a rough estimate of when the rapid population expansion began. Using a mutation rate of 2.8% per myr for the *C. angulata*^15^ COI, the time of demographic expansion for overall populations was estimated at ~91 Kya (95% CI: 35–138 Kya), for Chinese populations at ~118 Kya (95% CI: 33–196 Kya), and for Taiwanese populations at ~64 Kya (95% CI: 37–79 Kya). In contrast, a Bayesian skyline plot (BSP) showed that the initiation of overall *C. angulata* population growth was approximately 12,000 years ago and reached a stable effective population size approximately 3,000 years ago (Fig. 4a). When estimations were conducted on Taiwanese and Chinese populations, a BSP showed that Taiwanese *C. angulata* population growth began approximately 16,000 years ago and reached a stable effective population size approximately 4,000 years ago (Fig. 4b). In contrast, Chinese population growth began 6,000 years ago and reached a stable effective population size less than 1,000 years ago (Fig. 4c). In contrast, a BSP showed that *C. gigas* population grew and remained a stable effective population size before 45,000 years ago (Fig. 4d).

All the pairwise F_st_ values between *C. angulata* populations along the Chinese coast and 20 of 28 pairwise F_st_ between populations along the Taiwanese coast were not significant (Table 3). In contrast, 28 of 32 pairwise comparisons between Taiwanese and Chinese populations showed significant F_st_ values (*p* < 0.05). The overall population subdivision between Chinese and Taiwanese coasts was highly significantly different (F_st_ = 0.10167, *p < *0.001), suggesting a limited gene flow across the Taiwan Strait. An analysis of molecular variance (AMOVA) supported this tendency with a significant among-group Φ_CT_ (0.09787) between Taiwanese and Chinese coast populations, although 88.26% of the variance existed within populations (Supplementary Table 1S).

## Discussion

By intensively sampling cupped oyster populations island-wide, we confirmed that the *Crassostrea* species in Taiwan is *C. angulata* and that no *C. gigas* occurs in the natural environments of Taiwan and southern China sea waters. Several studies on the origin of *C. angulata* in Europe have used small numbers of *Crassostrea* previously identified as *C. gigas* from a single population in Taiwan as Asian references. Our result shows that *C. angulata* has an Asian origin and probably was introduced to Portugal from Taiwan in the 16th century^3^,^4^,^25^. Further surveys of cultured cupped oysters with five samples from Chiayi, Taiwan, identified *C. angulata* as the main species in the southern China, although it was suggested that *C. angulata* is a subspecies of *C. gigas* in this region^9^. Although the species/subspecies status between *C. gigas* and *C. angulata* remains unsettled as they can hybridize in hatcheries and in Southern Portugal^9^,^26^, 2.67% sequence divergence of COI fragment demonstrates that these two species are genetic distinct based on the criteria of interspecific divergence normally >2% for the standard DNA barcoding^22^. Indeed, species phylogeny generated by 31 Asian *Crassostrea* mitochondrial genomes from Chinese coast clearly demonstrates that *C. angulata* and *C. gigas* are closely related but independent species that probably diverged about 2.7 mya^15^,^16^ during the late Pliocene when sea levels and sea surface temperatures (SST) were higher and warmer but starting to drop during the onset of the “age of ice-ages” in the Pleistocene^27^. We hypothesized that the common ancestor of *C. angulata* and *C. gigas* might represent a species (resembling *C. angulata*) that was adapted to the warmer SSTs along the West Pacific coast in the late Pliocene. When glacial-interglacial cycles emerged during the Pleistocene, dramatic changes in the area and configuration of the coastline along the West Pacific^28^ might have isolated ancestor populations. Isolated populations in West Pacific high latitudes evolved into cold-adapted species resembling *C. gigas*. Haplotypic diversity, nucleotide diversity, and demographic analyses of the COI gene also supported *C. angulata* and *C. gigas* being distinct species and that *C. angulata* is an older lineage than *C. gigas. C. angulata* had twice as much haplotypic diversity (*h*: 0.882) and four times the nucleotide diversity (π: 0.00396) of *C. gigas (h*: 0.457 and π: 0.00097)^20^. While the median-joining network (MJN) of *C. angulata* was complex and star-like, being complicated by multiple connections and high-frequency internal haplotypes, the MJN of *C. gigas* from the northwestern Pacific was simple and star-like, which is the most common and widespread haplotype and the one that is typically assumed to be the most ancestral^24^. Paleoclimatic fluctuations could also account for the present-day distributions of *C. angulata* in the warm, subtropical southwestern Pacific and *C. gigas* in the cool, temperate northwestern Pacific, with the Yangtze River as a natural biogeographic barrier to separate not only these two cupped oyster species but also other coastal marine species along the Asian continent^8^,^9^,^19^,^21^.

Significant population expansion was identified for *C. angulata* along the coast of Taiwan and southern China based on different lines of evidence including network, mismatch distribution analysis (MDA), and neutral tests. For example, populations in Taiwan and southern China experienced different expansion models, with the former having a sudden population expansion and unimodal frequency distribution and the latter with a recent bottleneck or expansion and a negative binomial frequency distribution. The different expansion history of *C. angulata,* as indicated by MDA, is that the Chinese population expanded around 118 Kya and the Taiwanese population around 64 Kya. Beginning 140 Kya during the last interglacial period, Earth’s air temperature rose to approximately 2 °C above the present level and sea level rose to 5–6 meters or more above present ocean levels by 130–127 Kya^29^; therefore, ancestral *C. angulata* populations might have had the chance to colonize and expand along the coast of southern China as well as western Taiwan. However, this scenario was not supported because, following this interglacial high stand and several more oscillations, sea level dropped 120–130 m below the present level during the last glacial maximal (LGM; 21 Kya). Thus, *C. angulata* populations must be extinct because southern China and western Taiwan became connected when the sea level dropped during the LGM. In contrast, the Bayesian sky plot (BSP) matched the sequences of demographic histories for Taiwanese and southern Chinese populations to the effects of the LGM. That is, the BSP fits the time needed for their expansion to stable population sizes after another rapid rise in sea level (over a 12 Kya period) stabilized close to the present level 7,000 years ago^30^. However, caution should be taken in interpreting the effects of interglacial-glacial sea-level fluctuations on the population expansion history of *C. angulata* in this region because in consistent estimations of demographic expansion between MDA versus the BSP analysis have been noticed in several marine species in the marginal seas of the northwestern Pacific^19^,^21^. Methodological difference between MDA and BSP single-locus phylogeny using mtDNA and the uncertainty of the mtDNA mutation rate have been proposed to account for the estimation Bayesian determining expansion times^21^,^31^. Future multi-locus analyses (*e.g*., microsatellites, SNPs) should be considered for confirming the population expansion of *C. angulata* along the Taiwanese and southern Chinese coasts.

Although caution should be taken to interpret genetic connectivity based on single-locus mtDNA fragments such as the COI gene, the pairwise F_st_ between Taiwanese and southern Chinese populations of *C. angulata* is still highly significant, suggesting that cross-strait gene flow is limited. The Taiwan Strait, situated between the north end of the South China Sea (SCS) and south side of the East China Sea (ECS), has been recognized as a corridor for the migration of marine species between the two seas, aided by complex ocean currents around Taiwan (reviewed in ref. 18). Most studies of marine species in this region have focused on the phylogeography and connectivity between the SCS and ECS (reviewed in refs 19 and 21) and little attention has been paid to connectivity between the two sides of the Taiwan Strait. On the contrary, seasonal changes in northward and southward currents, such as the South China Sea Surface Current in the summer and China Coastal Water in the winter, might create a boundary that blocks cross-strait gene flow in *C. angulata.* A similar pattern was observed in the horseshoe crab, *Tachypleus tridentatus*, with gene flow being strong along the Chinese coast but limited in its ability to cross the Taiwan Strait^32^. Further studies using multi-locus population genetic analyses of *C. angulata* and more common benthic species should be done to confirm this observed pattern of restricted gene flow across the Taiwan Strait.

Confirmation of *C. angulata* in Taiwan is important not only to science but also to the oyster farming industry that plays a significant role in aquacultural economics^2^. For example, crosses between the Japanese *C. gigas* and Taiwanese cultivated *Crassostrea* and breeding of triploids have both been attempted but eventually failed in the last few decades in Taiwan^33^. Using standard COI DNA barcoding, we have demonstrated that the cultivated cupped oyster in Taiwan is *C. angulata,* not *C. gigas.* Thus, the failure to produce viable hybrid offspring from Japanese *C. gigas* and Taiwanese *C. angulata* is not unexpected even though natural hybrids between these two cupped oyster species have been detected by a microsatellite locus in southern Europe^7^. In addition, significant differences in demographic history, mtDNA genetic diversity, and limited gene flow across the Taiwan Strait highlight the importance of conserving the uniqueness of the Taiwanese *C. angulata* population, particularly under the current impacts of large-scale environmental disturbances that include marine pollution, habitat destruction, and climate change around Taiwan.

## Materials and Methods

### Ethics Statement

No specific permits were required for the described studies, and no specific permissions were required for these locations/activities.

### Sampling and DNA sequencing

In total, 313 individual oyster specimens were collected from 12 locations that were subdivided into two geographical regions (Taiwanese coast and Chinese coast) (Supplementary Fig. 1S; Supplementary Table 1S). Eight foreign (*e.g*., Japan, Korea, Australia, and America) *C. gigas* samples were purchased from importers and restaurants. Ten additional species of *Crassostrea* were selected as outgroups (COI: *C. virginica* EU007484, *C. cuttackensis* FJ262983, *C. belcheri* AY038077, *C. gryphoides* EU007491, *C. madrasensis* EU007462, *C. iredalei* AY038078, *C. hongkongensis* AY632557, *C. nippona* AF300616, *C. ariakensis* AY160752, *C. sikamea* EU007475; ITS-1: *C. virginica* EU072460, *C. rivularis* DQ785895, *C. ariakensis* FJ356683, *C. hongkongensis* EU073317, *C. nippona* FJ356681, *C. madrasensis* EU073241, *C. iredalei* EU073329, *C. gryphoides* EU073247, *C. belcheri* EU073259, and *C. sikamea* AB735523). Collected samples were frozen and transported to the laboratory. Adductor muscle tissue was cut into pieces and preserved in 95% alcohol. All reference samples are preserved in the Molecular Systematic Laboratory, Marine Fisheries Division, Fisheries Research Institute.

For initial species diagnosis, COI was selected as the molecular marker. Total genomic DNA was extracted from adductor muscle tissue using a commercial DNA isolation kit (Gentra, Minneapolis, MN, USA). The LCO-1490 (forward primer, 5′-GGT CAA CAA ATC ATA AAG ATA TTG G-3′) and HCO-2198 (reverse primer, 5′-TAA ACT TCA GGG TGA CCA AAA AAT CA-3′) primer set that amplifies COI was used^34^.The PCR reaction mixture included 5 μL of 10 × PCR buffer (Perkin-Elmer, Foster City, CA, USA), 4 μL of dNTPs (2.5 mM each), 2 μL of each primer (5 μM), 0.5 μL of 1.25-unit TaKaRa Taq (Takara Bio, Shiga, Japan), and 1.0 μL of a template containing approximately 5 ng of DNA; ddH_2_O was added to make a volume of 50 μL. A model 2400 thermal cycler (Perkin-Elmer) was used for PCR and conditions were set as follows: one cycle at 95 °C for 4 min, 40 cycles at 95 °C for 1 min, at 50 °C for 1 min, at 72 °C for 3 min, and a final elongation at 72 °C for 7 min. The reaction was stopped and products were preserved at 4 °C.

PCR products were examined on 1% agarose gel by electrolysis to confirm that the correct fragment length had been obtained, and then eluted using the QIAquick Gel Extraction kit (QIAGEN, Hilden, Germany). Purified PCR products were sent to Mission Biotech (Taipei, Taiwan) for DNA sequencing.

To reinforce the accuracy of species diagnosis and to determine whether *C. gigas* and hybrids (*C. gigas* × *C. angulata*) occur in Taiwan, we also sequenced the nuclear ITS-1 region with primers described by Hedgecock *et al.*^35^. We amplified the samples, which were haplotypes recognized from COI sequences, and used two or more replicates of each. The cycling conditions were one cycle at 95 °C for 4 min, 40 cycles at 95 °C for 1 min, at 55 °C for 1 min, at 72 °C for 3 min, and a final elongation at 72 °C for 7 min. PCR products were electrophoresed on 1% agarose gel to check their lengths. Amplified fragments of ITS-1 region were cloned into the yT&A cloning vector (Yeastern Biotech, Taipei, Taiwan) and transformed into *Escherichia coli* JM109 according the manufacturer’s protocol. Plasmids were screened for inserts and inserts were sent to Mission Biotech (Taipei, Taiwan) for DNA sequencing.

### Phylogenetic and haplotype network analyses

COI and ITS-1 sequences were aligned by ClustalW in MEGA 6.0^36^. DnaSP 5.10^37^ was used to identify haplotypes and polymorphic sites. Nucleotide sequence data for the haplotypes used in this paper were deposited in GenBank under accession numbers KU726888– KU726939. Maximum likelihood (ML) trees were reconstructed with a Kimura two-parameter model using MEGA 6.0 for both COI and ITS-1 sequences. The evaluation of statistical confidence was based on bootstrapping with 1,000 pseudo-replicates for ML^38^. Bayesian analysis was performed using MrBayes version 3.1.2^39^. The best-fit model of nucleotide substitution was the HKY + I model, which was selected using jModelTest version 2.1.7^40^. This model allows for a different rate of transitions and transversions and unequal frequencies of the four nucleotides, with a proportion of invariable site, I = 0.80. COI analyses were run with the best-fit model for 2.3 × 10^7^ generations and a sampling frequency of 100 generations (standard deviation of split frequencies = 0.008835), and 3 × 10^6^ generations for the ITS-1 analysis (standard deviation of split frequencies = 0.009508. Phylogenetic trees were visualized and edited with FigTree (http://tree.bio.ed.ac.uk/software/figtree/).

Gene diversity was described as haplotype diversity (*h*), endemic haplotypes (*eh*), and nucleotide diversity (*π*) for each population using DnaSP 5.10. Pairwise genetic divergence between populations was estimated by using Fst values and significance was verified through 10,000 permutations and adjusted by sequential Bonferroni correction^41^. Historical demographic history was obtained with neutrality tests, mismatch distributions, and a Bayesian Skyline Plot based on COI data. As for the neutrality test, Tajima’s D test^42^ and Fu’s Fs test^43^ were calculated using Arlequin 3.5^44^ with 10,000 permutations. Mismatch distribution was constructed for each geographic population to test a model of exponential population growth^45^. A goodness of fit test was performed to test the validity of the sudden expansion model using a parametric bootstrap approach based on the sum of square deviations (SSD) between observed and expected mismatch distributions. The demographic expansion parameter (τ) was calculated by Arlequin 3.5.

To detect any differences in genetic structure among populations using analysis of molecular variances (AMOVA), we grouped populations into three types: (a) those along Taiwanese (NTC, HC, CH, CY, TN, PT, KL, TT) and Chinese coasts (WZ, MT, KM, BH), (b) those along the Taiwanese (NTC, HC, CH, CY, TN, PT, KL, TT, MT, KM) and Chinese coasts(WZ, BH), and (c) those along Taiwan’s east (YL, TT) and west coasts (NTC, HC, CH, CY, TN, PT) and the China coast (WZ, MT, KM, BH). This first analysis was carried out to determine whether there were any differences in genetic structure across the Taiwan Strait. Oyster spats are regularly transported between Taiwanese coast and two islands, MT and KM, along the Chinese coast for marine culture^46^, therefore, the second analysis was carried out to examine whether the oyster spat transportation affected the genetic structure of oyster populations along the Chinese coast. The third analysis was carried out to determine whether there were any differences in genetic structure between Taiwan east coast and west coast populations. Pairwise genetic divergence between populations was estimated using the fixation index *Φ*st^47^. Demographic history and divergence time for the *C. angulata* COI sequence were further estimated using Bayesian evolutionary analysis sampling trees (BEAST) ver. 2.1.3^48^. Changes in effective population size (*Ne*) across time were inferred using Bayesian skyline analyses. To infer demographic history, coalescence methods require an initial demographic model to be specified. Where evidence of population expansion was found, the timing of expansion in generations (t) was estimated from τ = 2 μt, where τ (tau) is a parameter of the time to expansion in units of mutations and μ is the mutation rate per generation for the DNA sequence under study. The mutation rate of *C. angulata* was calculated as 2.785% per nucleotide per Myr since the divergence time was about 2.7 Myr for *C. angulata* and *C. gigas*^15^. The analysis was run with 50 million steps in a Markov chain Monte Carlo (MCMC) simulation under the HKY + I model, relaxed molecular clock model (uncorrelated log-normal). Other operators were optimized automatically. Results were viewed with Tracer v1.5^49^.

The haplotype network of the COI gene was inferred using the median-joining algorithm^50^. Before the calculation, the star contraction method with a maximum star radius value of 10 was used to simplify the data matrix. The MJN was drawn by Network, version 4.6.1.0 (Fluxus Technology, U.K.).

## Additional Information

**How to cite this article**: Hsiao, S.-T. *et al.* DNA barcoding reveals that the common cupped oyster in Taiwan is the Portuguese oyster *Crassostrea angulata* (Ostreoida; Ostreidae), not *C. gigas. Sci. Rep.* **6**, 34057; doi: 10.1038/srep34057 (2016).

## Supplementary Material

Supplementary Information

## Figures and Tables

**Table 1:** Sampling sites, sample size (*n*), number of endemic haplotypes (*n*_*eh*_), number of haplotypes (*n*_*h*_), haplotypic diversity (*h* ± SD), and nucleotide diversity (π ± SD) from samples of *Crassostrea angulata* collected from Taiwan.

Presumptive species ID	Locality		Code	*n*	*n*_*h*_	*n*_*eh*_	*h* ± SD	π ± SD
*Crassostrea angulata*	Taiwan coast	New Taipei City	NTC	24	10	10	0.804 ± 0.070	0.00282 ± 0.00062
		Hsinchu	HC	12	5	2	0.667 ± 0.141	0.00197 ± 0.00060
		Changhua	CH	25	8	3	0.850 ± 0.041	0.00314 ± 0.00047
		Chiayi	CY	42	14	8	0.847 ± 0.040	0.00319 ± 0.00032
		Tainan	TN	24	11	5	0.815 ± 0.063	0.00511 ± 0.00088
		Pingtung	PT	54	11	4	0.841 ± 0.025	0.00321 ± 0.00022
		Yilan	YL	22	11	3	0.896 ± 0.041	0.00315 ± 0.00043
		Taitung	TT	16	8	3	0.842 ± 0.075	0.00452 ± 0.00088
	China coast	Wenzhou	WZ	22	4	0	0.762 ± 0.040	0.00494 ± 0.00047
		Matzu	MT	28	7	0	0.791 ± 0.054	0.00404 ± 0.00054
		Kinmen	KM	21	12	5	0.886 ± 0.059	0.00448 ± 0.00071
		Beihai	BH	23	4	0	0.779 ± 0.031	0.00455 ± 0.00054
Total				313		43	0.852 ± 0.012	0.00396 ± 0.00017

**Table 2:** Demographic history summary statistics of tested *C. angulata* populations.

Population	Tajima’s *D*	Fu’s *F*s	Mismatch distributation					
			s	95% Cl Si	τ	θ^0^	θ^1^	P(SSD_obs_)
Chinese	0.01183	−1.11287	13	12–28	3.715(1.027–6.164)	0.005(0–0.877)	5.43823(3.13–99999)	0.014
Taiwanese	−2.03279*	−27.30487***	39	38–65	2.016(1.172–2.492)	0.021(0–0.636)	22.09531(5.96–99999)	0.001
Total	−1.01048	−14.20887	26		2.865(1.100–4.328)	0.013(0–0.757)	13.76677(4.54–99999)	0.007

^*^p < 0.05; ^**^P < 0.01; ^***^P < 0.001.

**Table 3:** Pairwise Fst values for population differentiation in *C. angulata*, as computed by Arlequin version 3.1, sampled from different sites in Taiwan.

Taiwan Coast									China Coast			
	NTC	HC	CH	CY	TN	PT	YL	TT	WZ	MT	KM	BH
NTC	—											
HC	−0.024	—										
CH	0.062*	0.119**	—									
CY	0.033*	0.052	−0.009	—								
TN	0.059*	0.077*	−0.006	0.006	—							
PT	0.084**	0.120*	−0.007	0.008	−0.005	—						
YL	0.030	0.084*	−0.019	−0.009	0.010	0.010	—					
TT	0.008	0.020	−0.001	−0.002	−0.006	0.040	0.008	—				
WZ	0.149*	0.166*	0.097*	0.117**	0.064*	0.151**	0.134*	0.017	—			
MT	0.247**	0.295**	0.083*	0.140**	0.068*	0.119**	0.147**	0.094*	0.047	—		
KM	0.222**	0.264**	0.054*	0.109**	0.049*	0.085**	0.113**	0.076*	0.061	−0.031	—	
BH	0.131**	0.144*	0.064	0.074*	0.047	0.110**	0.101*	0.004	−0.031	0.041	0.039	—

Abbreviations are listed in Table 1.

Significant Fst values ^*^(p < 0.05), ^**^(p < 0.01).
