# Supplementary material for: DNA barcoding reveals that the common cupped oyster in Taiwan is the Portuguese oyster Crassostrea angulata (Ostreoida; Ostreidae), not C. gigas
Source: Sci Rep. 2016 Sep 26;6:34057. doi: 10.1038/srep34057 (PMC5036096; doi:10.1038/srep34057)
Supplement: Supplementary Information [file srep34057-s1.pdf]

DNA barcoding resurrects the common cupped oyster as Portuguese oyster *Crassostrea angulata* (Ostreoida; Ostreidae) in Taiwan

Sheng-Tai Hsiao[1,2], Shin-Chang Chuang[1], Kao-Sung Chen[1], Ping-Ho Ho[2], Chi-Lun Wu[1], and Chaolun AllenChen [3,4,5*]

[1] Fisheries Research Institute, Council of Agriculture, Keelung, 20246, Taiwan

[2] Department of Environment Biology and Fisheries Science, Keelung, National Taiwan Ocean University, 20224, Taiwan

[3] Biodiversity Research Center, Academia Sinica, Nangang, Taipei, 11574, Taiwan

[4] Taiwan International Graduate Program-Biodiversity, Academia Sinica, Nangang, Taipei, 11574, Taiwan

[5] Institute of Oceanography, National Taiwan University, Taipei 10617, Taiwan

*Corresponding author: Chaolun Allen Chen, Biodiversity Research Center, Academia Sinica, Taiwan. TEL: 886-2-27899549; E-mail: cac@gate.sinica.edu.tw

Supplementary Figure legends

Fig. 1S.Sampling sites for Taiwan *Crassostrea* oysters. Map was generated using Surfer software (Surfer® [ver. 8] from Golden Software, LLC. (www.goldensoftware.com).

Fig. 2S.Phylogenetic tree using ITS haplotypes showing relationships of *crassostrea* species. ML bootstrap support for nodes were >70% and Bayesian posterior probabilities > 0.80, given below. Topology was identical with Bayesian (–lnL = 2967.47) and ML (–lnL = 1856.34) analyses.

Table 1S:Analysis of molecular variance (AMOVA) among *Crassostrea angulata* COI sequences based on Φ_{st}.

Hierarchical level	df	Variance component	Fixation indices	Percentage of variation	*P*
Geographic region (WZ, MT, KM, BH) (NTC, HC, CH, CY, TN, PT, YL, TT)					
Among groups	1	0.11536	*Φ*CT= 0.09787	9.79	0.00000*
Among populations within groups	10	0.02301	*Φ*SC= 0.02164	1.95	0.02835*
Within populations	301	1.04039	*Φ*ST= 0.11738	88.26	0.00391*
Geographic region (WZ, BH) (NTC, HC, CH, CY, TN, PT, YL, TT, MT, KM)					
Among groups	1	0.07929	*Φ*CT= 0.06749	6.75	0.05963*
Among populations within groups	10	0.05511	*Φ*SC= 0.05030	4.69	0.00000*
Within populations	301	1.04039	*Φ*ST= 0.11440	88.56	0.00000*
Geographic region (WZ, MT, KM, BH) (NTC, HC, CH, CY, TN, PT) (YL, TT)					
Among groups	2	0.08122	*Φ*CT= 0.07076	7.08	0.00196*
Among populations within groups	9	0.02627	*Φ*SC= 0.02463	2.29	0.01760*
Within populations	301	1.04039	*Φ*ST= 0.09364	90.64	0.00000*

Abbreviations are listed in Table 1

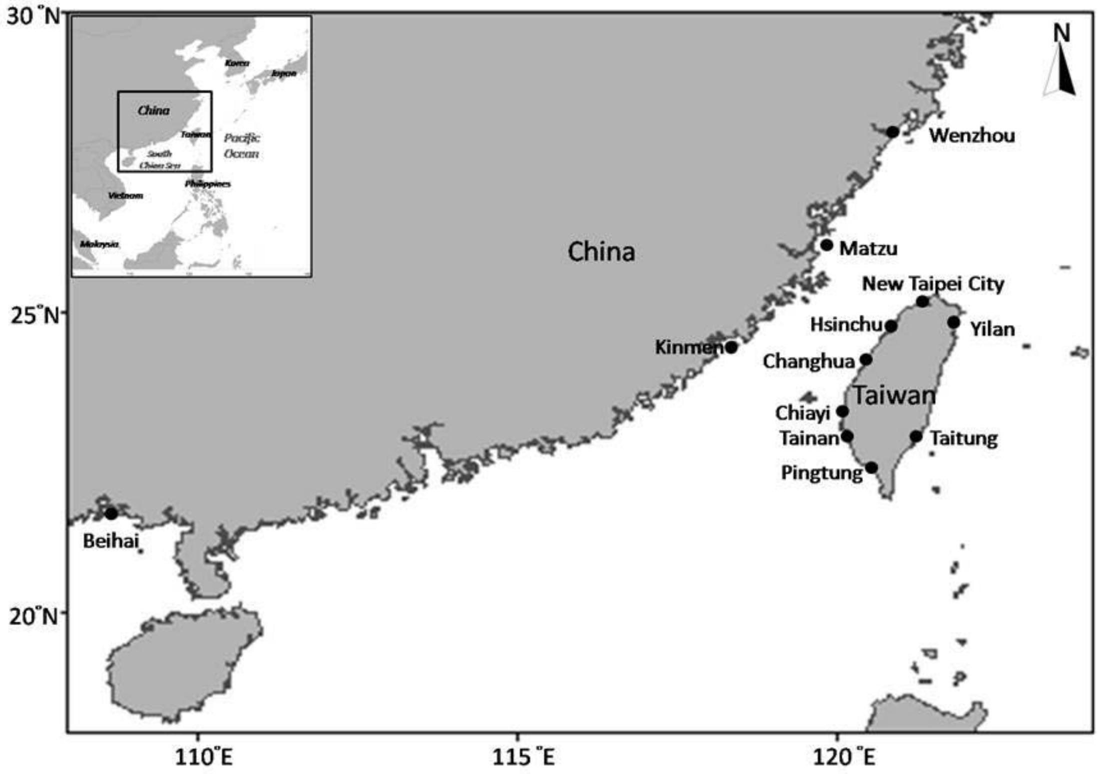
30˚N
25˚N
20˚N
110˚E
115˚E
120˚E
N
China
Taiwan
Wenzhou
Matzu
New Taipei City
Hsinchu
Yilan
Kinmen
Changhua
Chiayi
Tainan
Taitung
Pingtung
Beihai
Korea
Japan
China
Taiwan
Pacific
Ocean
South
China Sea
Philippines
Vietnam
Malaysia

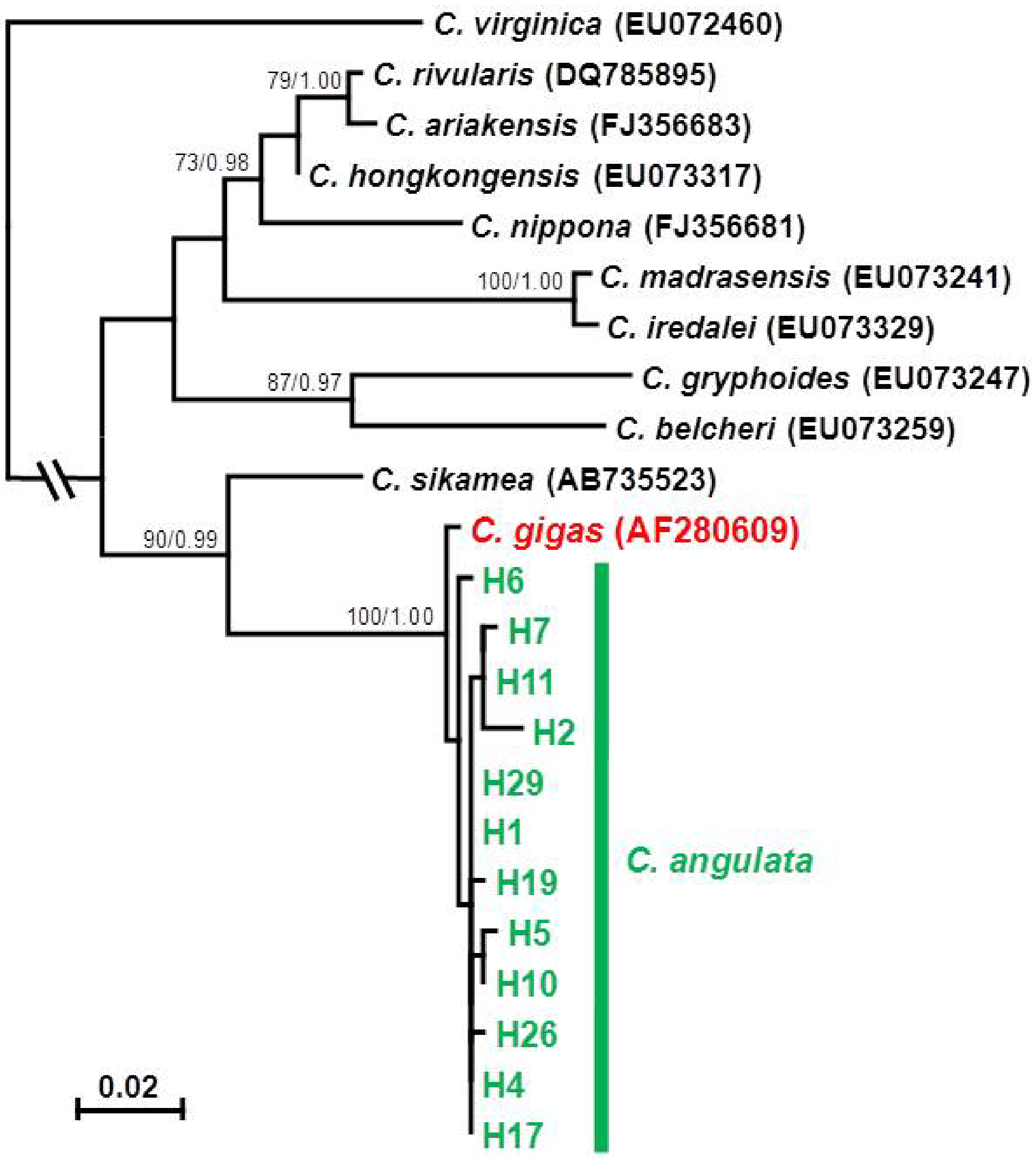
C. virginica (EU072460)
79/1.00
C. rivularis (DQ785895)
C. ariakensis (FJ356683)
73/0.98
C. hongkongensis (EU073317)
C. nippona (FJ356681)
100/1.00
C. madrasensis (EU073241)
C. iredalei (EU073329)
87/0.97
C. gryphoides (EU073247)
C. belcheri (EU073259)
C. sikamea (AB735523)
90/0.99
C. gigas (AF280609)
100/1.00
H6
H7
H11
H2
H29
H1
H19
H5
H10
H26
H4
H17
C. angulata
0.02
